# Nano-Electrochemical Characterization of a 3D Bioprinted Cervical Tumor Model

**DOI:** 10.3390/cancers15041327

**Published:** 2023-02-19

**Authors:** Maila Becconi, Simona De Zio, Francesco Falciani, Marzia Santamaria, Marco Malferrari, Stefania Rapino

**Affiliations:** Department of Chemistry “Giacomo Ciamician”, University of Bologna, Via Francesco Selmi 2, 40126 Bologna, Italy

**Keywords:** scanning electrochemical microscopy, 3D bioprinting, cervical tumor model, hypoxia, cellular microenvironment

## Abstract

**Simple Summary:**

3D bioprinting has been shown to be an extremely useful method for the fabrication of in vitro tridimensional cellular models which better resemble the structural and functional complexity of real tissues. The cell microenvironment is a crucial determinant of cell behavior and its recreation in cellular in vitro models is fundamental to performing reliable biomedical and biotechnological experimentations. In this work, we employed extrusion 3D bioprinting using a bioink containing HeLa cells to build a model of a cervical tumor; the resulting HeLa spheroids were described in terms of their dimensions and expression of membrane proteins involved in cell adhesion. A key cellular feature of the microenvironment—the oxygen concentration within HeLa spheroids—was determined by scanning electrochemical microscopy with a micrometric spatial resolution using platinum nanoelectrodes. Scanning electrochemical microscopy was also employed to study the diffusion of a molecule in the biofabricated cervical tumor construct, as a model of drug diffusion in the 3D architecture.

**Abstract:**

Current cancer research is limited by the availability of reliable in vivo and in vitro models that are able to reproduce the fundamental hallmarks of cancer. Animal experimentation is of paramount importance in the progress of research, but it is becoming more evident that it has several limitations due to the numerous differences between animal tissues and real, in vivo human tissues. 3D bioprinting techniques have become an attractive tool for many basic and applied research fields. Concerning cancer, this technology has enabled the development of three-dimensional in vitro tumor models that recreate the characteristics of real tissues and look extremely promising for studying cancer cell biology. As 3D bioprinting is a relatively recently developed technique, there is still a lack of characterization of the chemical cellular microenvironment of 3D bioprinted constructs. In this work, we fabricated a cervical tumor model obtained by 3D bioprinting of HeLa cells in an alginate-based matrix. Characterization of the spheroid population obtained as a function of culturing time was performed by phase-contrast and confocal fluorescence microscopies. Scanning electrochemical microscopy and platinum nanoelectrodes were employed to characterize oxygen concentrations—a fundamental characteristic of the cellular microenvironment—with a high spatial resolution within the 3D bioprinted cervical tumor model; we also demonstrated that the diffusion of a molecular model of drugs in the 3D bioprinted construct, in which the spheroids were embedded, could be measured quantitatively over time using scanning electrochemical microscopy.

## 1. Introduction

In the past years, the metabolism of cancer cells has emerged to be an important factor for determining resistance to therapies and disease relapse [1,2,3,4]. Metabolic adaptations maximize growing rate in the specific conditions established in the tumor mass microenvironment. The cell microenvironment is defined by the activity of the surrounding cells and by a chemical component, both in relation to the three-dimensional architecture of the tumor masses. Gradients of nutrients and metabolites (e.g., oxygen and glucose) in the masses and the different positions of the cell types with respect to those gradients are just two of the many levels of complexity that contribute to an established cancer cell microenvironment [5,6].

Over the past fifty years, the scientific community has concluded that 2D cell cultures are not fully representative of the conditions that cells experience in tissues, as many features are altered in 2D versus 3D cultures [7,8,9,10]; this is probably one of the factors that mainly determines the differences which are evidenced in the results of in vitro tests with respect to the subsequent phases of drug development and selection, from in vivo to clinical phases. The structural and dynamical properties of the 3D environment influence molecular diffusion, generate concentration gradients of the soluble factors, and mediate cell response to external mechanical inputs [11,12]. For this reason, cells cultured in a 3D fashion give different responses to drugs, different gene expression, and a different degree of cell differentiation compared with 2D cell culture [7]. When embedded in matrices enabling 3D cultures or when adhesion is prevented, cells originate several types of aggregate or pluricellular structures [13], which have been investigated for their ability to reproduce characteristics of tissues or, concerning cancer, of tumor masses. Among these structures, spheroids have been shown to be obtainable with numerous cell types and to be able to effectively reproduce some key characteristics of real tumor tissues. 

Tumor spheroids are spheroidal-shaped cell clusters with a variable diameter, from ~100 µm to more than 500 µm. They originate from the duplications of a single cell under specific experimental conditions. Inside a spheroid, several pathophysiological gradients are established, usually with a concentric arrangement: the oxygen content, for example, is thought to decrease moving towards the core of the spheroid; as a result, several cells are differently arranged with respect to the direction of the oxygen gradients: from the external to the internal part, respectively, migratory cells, proliferating cells, quiescent live cells, apoptotic cells, and necrotic cells (concentrated in the more hypoxic core) are found [14]. Relevantly, oxygen concentration is one of the main regulators of cellular processes and a key player in cellular behavior in physiological and pathological conditions [1,2,15,16,17].

When spheroids are embedded in hydrogels, different cell–cell and cell–matrix interactions and adhesions, different orientations of the fibrils that make up the extracellular matrix, and variations in the hardness of the surrounding matrix, are observed [7,10]. Production of well-defined 3D systems is a big challenge, requiring not only control of the cell microenvironment, but also scaffold nano-porosity, growth factor-binding, and matrix degradation [18]. The extracellular matrix (ECM) is a very versatile structure; it influences cell–matrix and cell–cell interactions and undergoes constant remodeling. The cells themselves secrete ECM molecules in order to create suitable, stable surroundings in which cells can proliferate [19,20]. ECM stiffness has a fundamental role in cancer progression, and the mechanotransduction of external physical cues regulates cellular pathways and signaling [21]. When cancer occurs, cancer cells secrete different ECM components and ECM-modifying enzymes in order to alter the tumor stroma, both qualitatively and quantitatively, affecting the adhesion, motility, differentiation, proliferation, and apoptosis of the cells [19,20,22]. 

In the present work, we aimed to develop a methodology for obtaining and characterizing, with high reproducibility, tridimensional cervical tumor models, which are distinguished by defined gradients of chemical components in the cell microenvironment; HeLa cells were employed, being one of the most frequently utilized immortalized cervical cancer cell lines. 

We 3D-printed cell-embedded hydrogels, cultured the 3D models for up to 40 days, and characterized the resulting spheroids for their morphology, dimensions, and cellular organization during this time. We also characterized the expression of cadherins, membrane protein complexes directly involved in cellular adhesion, in the obtained 3D models. Three-dimensional bioprinting has been shown to be an extremely powerful approach for developing 3D cell cultures constituted by different cell types, arranged in a precise and controlled fashion in space, with a sub-millimetric resolution [23]. Several 3D-bioprinting methodologies have been proposed, such as: extrusion bioprinting [24], inkjet bioprinting [25], and VAT polymerization [26]. In the present work, extrusion bioprinting was chosen in view of the good cell viability usually observed by employing this printing technique [27,28].

We pioneered the use of nanoelectrodes and scanning electrochemical microscopy (SECM) to characterize the cell microenvironment within the 3D bioprinted tumor models. SECM has been largely employed to investigate bidimensional cell cultures and tissues [29,30,31,32,33,34,35,36]: studies of cellular metabolism [31,37,38] and at the single-cell level [32,33,39,40] have been conducted; other cellular characteristics have also been investigated, e.g., shape and adhesion [30]. In the past years, the development of nanoelectrodes has enabled the investigation of the intracellular subdomain without relevantly affecting cell viability and without perturbing the intracellular concentration of the species of interest [41,42,43]. SECM nanoelectrodes have been developed to explore both extracellular and intracellular nanodomains in bidimensional cell cultures, to detect molecules that are spatially confined or at low concentrations. For example, nanoelectrodes have enabled the investigation of reactive oxygen species (ROS) and reactive nitrogen species (RNS) content in cells and their effects on the overall cell redox balance [38,44]; these parameters are correlated to several pathological and physiological conditions of living cells. We aim to show the capability of SECM in sensing cellular metabolic activity within 3D cellular structures. In particular, oxygen consumption and the relative gradients generated within a single spheroid was investigated by employing SECM with nanometric probes without affecting the tridimensional structures. Ultramicroelectrodes have already been employed to study oxygen consumption rate by sensing oxygen in the proximity of spheroids [45,46]. In the present work, we employed nanoelectrodes as minimally invasive SECM probes [29,41,47,48] (i.e., the spheroids were not destroyed by the measurements), to quantify the concentration gradients that were present inside spheroids that were grown in a 3D bioprinted matrix. Additionally, by employing SECM we analyzed how diffusion processes occur within the three-dimensional bioprinted cervical tumor constructs by evaluating the penetration and diffusion of the molecules in the 3D environment at a high temporal resolution (i.e., milliseconds). This information will be used in the future to guide the design of 3D bioprinted models mimicking the diffusion of anticancer compounds in the tumor mass. 

## 2. Materials and Methods

### 2.1. Bioink Preparation and Optimization

Formulation of optimized bioink was 2.5% *w*/*v* alginate, 3.3% *w*/*v* mannitol, and 0.19% *w*/*v* calcium chloride in water. Sodium alginate from brown algae (W201502), calcium chloride (CaCl_2_) (C8106) and D-mannitol (M4125) were purchased from Sigma Aldrich (St. Louis, MO, USA). Aqueous 0.67% *w*/*v* calcium chloride solution was prepared by dissolving CaCl_2_ powder in distilled water and then sterilized with an autoclave. Alginate hydrogel was prepared by dissolving 3.5 % *w*/*v* of sodium alginate powder and 4.6% *w*/*v* of mannitol powder in distilled water and stirred for 5 h at room temperature (RT). Specific weight/volume values are determinants, as the density of bioprinted matrices is known to strongly influence the bioprinting process and the hydration state of the molecular components of the matrix, e.g., sugars or proteins [24,49,50]. The solution was sterilized in an autoclave for 20 min at 120 °C; just after temperature equilibration at RT, pre-crosslinking with calcium chloride solution in a ratio 25:10 = vol (Alginate):vol (calcium chloride) was performed [51].

### 2.2. Cell Cultures 

HeLa cells (ATCC number CCL-2) were employed in all cellular experiments. Both bidimensional and tridimensional cultures were maintained in Dulbecco’s Modified Eagle’s Medium (Microgem Naples, Italy, TL1006) supplemented with 10% Fetal Bovine Serum (Microgem, RM10432), 2 mM L-glutamine, 50 U/mL penicillin, 50 µg/mL streptomycin (Microgem) and cultured at 37 °C, 5% CO_2_. Bidimensional cultures were passed upon trypsin digestion every two or three days; the growth medium of 3D constructs was changed regularly, with a fresh one every two or three days.

### 2.3. Biofabrication of the Cervical Tumor Model

The procedure of bioink formulation includes a sterilizing step, involving autoclaving for 20 min at 120 °C. As high temperatures affect crosslinking, two distinct solutions were prepared separately and mixed after sterilization. The first solution was composed of 3.5% *w*/*v* alginate and 4.6% *w*/*v* D-Mannitol; the second one was 0.67% *w*/*v* CaCl_2_ in bidistilled water. The mixing was performed with a ratio 25:10 = solution 1:solution 2. The formulation of the final sterile bioink used in this work was 2.5% *w*/*v* alginate, 3.3% *w*/*v* D-Mannitol, 0.19% *w*/*v* CaCl_2_ in bidistilled water. The good printability characteristics of the final formulation are shown in Figure S1 (Supplementary Material). 

In this work, we employed a Cellink^®^ Inkredible+ 3D Bioprinter from the BICO Company (Gothenburg, Sweden). For each composition, the optimum printing parameters were defined by printing tests; as expected, the printability of alginate solutions depended on alginate molecular weight and crosslinking ratio [51]. High reproducibility in the structural performance of new 3D bioprinted models and high resolution in printing were achieved. Printability is defined herein by a printability index *Pr* [52]; high geometric accuracy result in a *Pr* = 1, while *Pr* < 1 corresponds to a round-shaped transversal geometry and *Pr* > 1 to an irregular-shaped transversal geometry. Filament uniformity is the fidelity of filament geometry and is a predictor of shape fidelity. 

**Figure 1:**
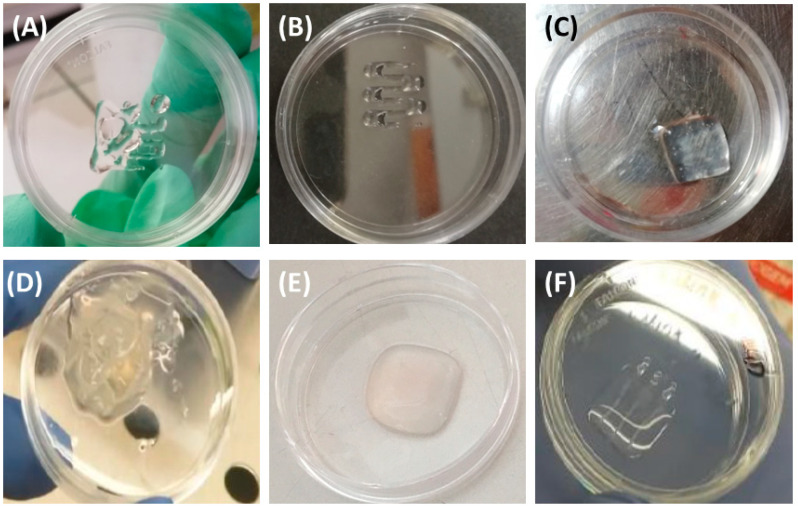
Alginate bioink optimization. (**A**) Printed grid of alginate bioink with a ratio between alginate solution of 3.5% *w*/*v* and 0.67% *w*/*v* CaCl_2_ equal to 25:9, *Pr* > 1. (**B**) Printed half-grid of alginate bioink with ratio 25:10, *Pr* < 1. (**C**) Cube of (1 × 1 × 0.15) cm^3^ made of alginate bioink before the crosslinking process. (**D**) Cube of (1 × 1 × 0.15) cm^3^ made of alginate bioink without D-mannitol and following the crosslinking process with 0.67% *w*/*v* CaCl_2_. (**E**) Cube of (1 × 1 × 0.15) cm^3^ made of alginate bioink with D-mannitol after the crosslinking process with 0.67% *w*/*v* CaCl_2_. (**F**) 3D bioprinted sample of cervical tumor model: a cube of (1 × 1 × 0.15) cm^3^ made of alginate bioink, inside of which are three printed lines of the bioink containing HeLa cells.

Different solution 1: solution 2 ratios were tested. The bioink obtained with a ratio of 25:8 was printed at P~15 kPa and resulted in a bioink with lower filament uniformity than the 25:9 formulation, which showed a printability ratio *Pr* > 1 and lower shape fidelity; for this reason, these formulations were not suitable for printing. Bioink obtained with a 25:11 ratio was printed with P~28 kPa and resulted in a clumpy and non-uniform filament which was difficult to extrude. The bioink characterized by a 25:10 ratio printed at P = 25 kPa, in spite of round-shaped transversal geometry, i.e., *Pr* < 1 (Figure 1B), resulting in a bioink with good filament uniformity and good extrusion homogeneity. This latter ratio resulted in the best performing bioink.

A cube-shaped construct with dimensions of (10 × 10 × 1.2) mm^3^ was printed with the bioink prepared according to the protocol described in Section 2.1; the 3D model used for the printing was released from Cellink template models included in the printer. Once the cubic scaffold was printed, three lines were added inside it, using a cellular suspension in the bioink. Cells harvested from standard cell cultures were harvested at a concentration of 10^6^ cells/mL in culture medium and mixed with the alginate bioink in a 4:1 ratio (alginate bioink/HeLa suspension). Crosslinking of the bioprinted model was made by pipetting 0.67% *w*/*v* calcium chloride solution on the construct and incubating for 5 min at RT; after quick washing with phosphate buffered saline (PBS) with calcium and magnesium (Gibco—part of ThermoFisher, Paisley, United Kingdom, 4040091), the HeLa 3D bioprinted construct was placed in a 35 mm diameter Petri dish and immersed in culture medium. Alginate printing conditions were settled as follows: layer height, 0.4 mm; printing temperature, RT; printing pressure, 20–30 kPa; diameter tip, 25 G; printing speed, 10 mm s^−1^. Cell printing took place at 26 °C using a plastic tip with a diameter of 27 G at a pressure of 15 kPa. The printing conditions were optimized to allow an optimal printing process, considering the different viscosities of the bioinks in the presence or absence of cells.

Models for 3D bioprinting were designed with Fusion360 (Autodesk) software (v.2.0, Autodesk, San Rafael, CA, USA); the procedure code for the printing process was elaborated by employing the open-source slicing software Slic3r (v.1.2.9,open source). For 3D bioprinting of the construct of Figure 1B,C,E we used a grid model available with the Inkredible+ printer (BICO Company, Gothenburg, Sweden). We bioprinted an ink containing cells in the tissue model scaffold of the alginate hydrogel, following a specific design: three lines of hydrogel containing cells with a 0.6 mm width and 3 mm distant from each other were printed in the (10 mm × 10 mm) cell-free scaffold at a depth of 0.4 mm from the bottom (Figure 1F). This design allows for high control and reproducibility in the diffusion and availability of nutrients in the 3D scaffold; such a controlled geometry cannot be achieved by manual casting. Moreover, 3D bioprinting assures high reproducibility on the amount of extruded material and the number of cells in the final construct.

### 2.4. Cell Viability Assays

**Figure 2:**
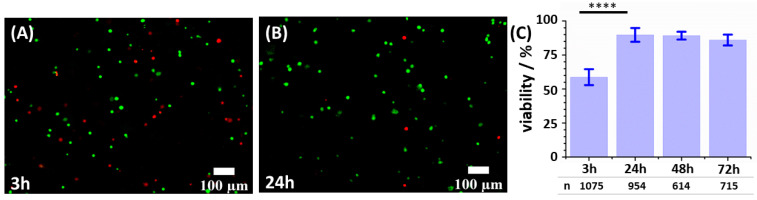
Live/dead assay with calcein-AM/propidium iodide. (**A**,**B**) Living cells (green, calcein-AM) and dead cells (red, propidium iodide) in a 3D bioprinted cervical tumor model at 3 h (**A**) and 24 h (**B**) after printing. (**C**) Viability of 3D bioprinted HeLa cells at 3 h, 24 h, 48 h, and 72 h after printing. Population size, *n*, i.e., the number of cells evaluated for each time point, is reported for each bar of the graph. Levels of statistical significance are calculated as detailed in Section 2.9 and are indicated as follow: ****, *p* < 0.0001.

To assess the biocompatibility of the formulated hydrogel and to evaluate how much the printing procedure affects cell viability, live/dead assays with calcein-AM (Thermofisher, Waltham, MA, USA, c1430) and propidium iodide (Merck, Rahway, NJ, USA, P4864) were performed on 3D bioprinted constructs cultured for 3, 24, 48, and 72 h. Briefly, the 3D bioprinted samples were washed with PBS with calcium and magnesium three times for 5 min, followed by incubation for 30 min at 37 °C with a solution of 3 µM calcein-AM and 4 µM propidium iodide in HBSS (Sigma H8264); before inspection with an optical microscope, the constructs were washed three times for 5 min with PBS with calcium and magnesium. The samples were examined with an inverted fluorescence microscope (Nikon eclipse TiS, Nikon instruments, Melville, NY, USA) using a 10× objective and by using two fluorescent channels; propidium iodide signals were measured with a Nikon Texas Red HYQ cubic filter (λ excitation = 532–587 nm, λ emission = 608–683 nm), while calcein-AM fluorescence was recorded by employing a Nikon FITC standard cubic filter (λ excitation = 465.0–495.0 nm, λ emission = 512.5–557.5 nm). At least eight random fields of view were taken for each sample. Viability percentages were estimated from the fluorescence images with a semi-quantitative analysis using Fiji software [53]: live cells (stained green with calcein-AM) and dead cells (stained red with propidium iodide) were manually counted and live cell fractions calculated by dividing the number of live cells by the total number of cells. Population size, *n*, i.e., the number of cells considered for each time point, is reported in Figure 2C: 3 h, *n* = 1075; 24 h, *n* = 954; 48 h, *n* = 614; 72 h, *n* = 715.

### 2.5. Measurements of Spheroid Dimensions and Morphologies

During the whole experimental process, a phase-contrast microscope (Nikon Ti-S) was used to observe spheroid morphology and measure their diameters. Bioprinted constructs were studied by monitoring them over time and by measuring the size of the spheroids; samples were monitored starting from day 5, when they became populated by spheroids with diameters not higher than 30 µm, to five weeks (day 34), when the samples start to be highly cellularized and analysis became much more difficult. Spheroid diameters were classified within the following ranges: 40–70 μm, 71–100 μm, 101–130 μm, 131–160 μm, 161–190 μm, 191–220 μm, and >221 μm. For each group, frequencies were calculated by dividing the number of spheroids in a specific range of diameters by the total number of spheroids counted in that experiment. 

Cellular morphology in 3D and 2D HeLa samples was evaluated by staining f-actin filaments and nuclei. Samples were treated as follows: washing three times with PBS with calcium and magnesium; fixing with 4% paraformaldehyde for 30 min; permeabilization with 0.1% Triton X-100 for 30 min; incubation with 1% bovine serum albumin (BSA) for 30 min; staining with 5 μg mL^−1^ of FITC-phalloidin (product number P5282, Sigma Adrich) for 20 min at room temperature in the dark; and staining with 1 μg mL^−1^ of 4′,6-diamidino-2-phenylindole (DAPI) for 10 min at room temperature in the dark. Incubation with primary anti-mouse antibody for E-cadherin (Santa Cruz, Dallas, TX, USA; sc-8426) was performed overnight at 4 °C; the antibody preparation was then diluted 1:400 in 2% BSA. The secondary anti-mouse goat antibody of Invitrogen Alexa-Fluor 568 was diluted 1:1000 in 2% BSA and incubated for 4 h at room temperature with the sample. Between each incubation, samples were washed three times for five minutes with PBS with calcium and magnesium.

For histological analysis, the 3D bioprinted samples were washed with 0.89% *w*/*v* CaCl_2_ solution for 5 min and then immersed in 70% *v*/*v* EtOH overnight at RT. Samples were embedded in paraffin and sectioned in slices of 4 µm of thickness. The sections were stained with hematoxylin–eosin (HE) and observed via optical microscopy with Leica ICC50W (Leica microsystem, Wetzlar, Germany).

### 2.6. Confocal Laser Scanning Microscopy 

Confocal laser scanning microscopy analyses were carried out on 3D bioprinted samples plated in 35 mm diameter glass-bottomed microwell dishes (Mattek, BICO Company, Ashland, MA, USA). 

Samples stained for viability assay were observed with a Nikon A1R confocal laser scanning microscope using two laser lines (489 nm and 561 nm) and two detection channels (525/50 and 595/50 nm) for the green and red false color channels, employed to measure the brightness of calcein-AM and propidium iodide, respectively. Viability percentages were estimated with a semi-quantitative analysis using Fiji software (v.1.52n, ImageJ, opensource) [53]: living cells (stained green with calcein-AM) and dead cells (stained red with propidium iodide) were manually counted and live cell fraction calculated by dividing the number of living cells by the total number of cells. 

Samples stained for morphological assay were observed with a Nikon A1R confocal laser scanning microscope using three laser lines (401 nm, 489 nm, and 561 nm) and three detection channels (450/50 nm, 525/50 nm, and 595/50 nm) for the blue, green and red false color channels, employed to measure, respectively, the brightness of DAPI, phalloidin-FITC, and the marker of interest (E-cadherin).

### 2.7. Scanning Electrochemical Microscopy

SECM measurements of oxygen concentration inside cancer cell spheroids and ferrocenemethanol diffusion in the bioprinted constructs were performed with a 910 B SECM instrument (CH Instruments) coupled with a Nikon *Ti* inverted fluorescence microscope; this modification was accomplished in-house [54]. The stepper motors and the piezoelectric component of the 910 B CHI instrument for the microelectrode displacement were removed from the original stage and mounted on the plate of the inverted microscope. All electrochemical measurements were carried out in Petri dishes located on the plate holder of the inverted microscope. The SECM probes were 10 µm diameter Pt UME with RG = 10. The tips were polished and cleaned prior to use with alumina-containing polishing cloths (0.05 µm) followed by sonication in a bath sonicator. A platinum wire was used as the counter electrode and all the potentials are referred to the (Ag/AgCl/3M KCl) reference electrode or quasi reference Ag wire. The dissolved oxygen was used as a redox mediator, and the UME was positioned over the plastic dish at a controlled tip/Petri dish distance estimated by the SECM approach curve at E_UME_ = −0.7 V vs. the quasi-reference Ag wire. The negative feedback resulting from the redox mediator hindrance due to the Petri surface was employed to precisely measure the distance from the dish surface. 

For the quantification of diffusion constant in the bioprinted construct, we used a 10 µm diameter Pt UME as the working electrode, Ag wire as the quasi-reference electrode, and Pt wire as the counter electrode. Ferrocenemethanol, FeMeOH, 97%, was purchased from Sigma Aldrich (product number 335061); 1.5 mM FeMeOH was obtained by dissolving FeMeOH powder in PBS and then sonicated until complete dissolution. At first, we performed cyclic voltammetry in the bulk outside the spheroids. Then we positioned the electrode inside the 3D matrix at 800 μm from the nearest construct/solution boundary; we performed cyclic voltammetry every 30 s for six hours. Voltammetries were performed from 0 to 0.6 V vs. Ag/AgCl/KCl 3M with a scan rate of 0.02 V/s. Schematic representation of SECM detection of the FeMeOH diffusion into the 3D bioprinted construct is reported in Figure S2.

### 2.8. Nanoelectrochemical Measurements of Oxygen Concentration Inside Spheroids

Electrochemical detection of O_2_ inside cancer cell spheroids was investigated using a 200–500 nm Pt nanoelectrode (Pt purity 99.99%) from Sensolytics GmbH (Bochum, Germany; Product Number 04-00006); the commercial electrode was modified by etching with fluoridric acid for 5–10 min to erode the non-active glass part and obtain a thinner tip. This procedure was adopted so as to not affect spheroid architecture during its penetration. Prior to perform the measurements, the nanoelectrode was cleaned by bathing it in sulphonitric solution for 5 min and then washing with distilled water. All measurements were made by immersing the scaffolds in PBS with calcium and magnesium. A potential of −0.65 V vs. the quasi-reference Ag wire was applied at the Pt nanoelectrode to electrochemically reduce O_2_; the resulting currents were proportional to the amount of local O_2_ concentration. A platinum wire was used as the counter electrode. Spheroid penetrations in the Z-axis were made with Probe Scan Curves (PSC) which allowed the measurement of currents at a constant potential as the electrode moved downwards. 

### 2.9. Statistical Analysis

Live/dead experiments were performed in three independent experiments, with the samples showing comparable viability among replicates; data shown in Figure 2 belong to one of the three replicates. Cell counts are represented as mean and errors as standard deviations. Statistical analyses were performed using OriginPro 9.1 software (Origin 2019b, OriginLab corporation, Northampton, MA, USA). Statistical significance was determined by two sample *t*-test. Differences were considered statistically significant at *p* < 0.05. Different levels of statistical significance are indicated as follow: *, *p* < 0.05; **, *p* < 0.01; ***, *p* < 0.001; ****, *p* < 0.0001.

## 3. Results and Discussion

### 3.1. Bioink: Formulation and Optimization

One of the most challenging step in the 3D bioprinting process is the development of proper bioinks [28]. They must fulfil a lot of requisites: biocompatibility; appropriate viscosity; a good balance between extrudability (to avoid printhead clogging) and good shape fidelity of bioprinted constructs; proper chemical and structural properties, which promote physiological cell behavior; and sufficient strength and stiffness, to maintain integrity after deposition [55]. Additionally, bioinks need to account for their peculiar properties to overcome issues arising with specific printing techniques, e.g. droplet satellite formation with inkjet printing [56]. We developed and optimized a printable hydrogel formulation suitable for extrusion bioprinting of HeLa cells and their successive culturing. As one of the main goals of this work was the development and demonstration of the characterization of 3D bioprinted tumor models, alginate was chosen among the variety of examples of biomaterials that are reported in literature for the 3D bioprinting of tumor models, such as chitosan-alginate, polyethylene glycol, and hyaluronic acid [57,58,59,60,61]. Alginate was chosen as the main constituent of the employed bioink, for the following reasons: (i) alginate is one of the most used hydrogels in bioprinting applications; (ii) it is highly biocompatible; (iii) it is a low-cost material; and (iv) a variety of cross-linking methods and bioprinting methodologies can be used and have been already tested for this hydrogel [28,62].

Usually, a pre-crosslinking step is mandatory to obtain an alginate bioink that is suitable for extrusion, due to the low viscosity of the alginate solution. To find the best ionic pre-crosslinking procedure, we tested several ratios of alginate/CaCl_2_, to obtain a bioink with a printability ratio less than or equal to 1 (*Pr* ≤ 1), which usually defines good shape fidelity and extrudability. 

In a first step, we tested pre-crosslinked alginate hydrogels by mixing alginate solution at 3.5% *w*/*v* and 0.67% *w*/*v* CaCl_2_ with ratios of 4:3 and 25:9. The two bioinks result in extremely different printabilities. The bioink characterized by the 4:3 ratio (higher calcium chloride concentration) does not allow a continuous extrusion (at printing pressure, P, of ~30 kPa, lumpy pieces of the filament were extruded). For this reason, this formulation was not suitable for 3D printing. The second bioink characterized with the 25:9 ratio held filament homogeneity while printing, reflecting a good extrudability, but showed low filament uniformity and irregularly shaped transversal geometry (i.e., *Pr* > 1) (Figure 1A). These results were consistent with the employed alginate, which behaved like a high-molecular-weight alginate, as reported previously [51]. 

We further optimized this bioink, evaluating different compositions of alginate/CaCl_2_ crosslinking agent in a range of ratios close to the 25:9 ratio, namely: 25:8; 25:10 (i.e., 5:2) and 25:11. We obtained three different alginate bioinks with different properties and printabilities; among the three compositions tested, the 25:10 ratio resulted to be extremely suitable for 3D bioprinting (see the Materials and Methods section for a detailed description of the bioprinting characteristics of the different ratio tested), resulting in a bioink with good filament uniformity and good extrusion homogeneity. This formulation was perfectly suitable for our purpose, i.e., to print a 1 cm × 1 cm full cube of alginate with 1.5 mm height (Figure 1C). However, printing of the construct (Figure 1C) and successive crosslinking by pipetting 0.67% *w*/*v* CaCl_2_ solution on the sample (see Materials and Methods section for details) resulted in samples that lost their shape in culturing conditions (37 °C, 5% CO_2_; Figure 1D). To overcome this problem, 4.6% of D-mannitol was added; D-mannitol’s ability to establish hydrogen bond networks gave stability to the crosslinked alginate chains, allowing the crosslinking process without losing the shape of the printed sample (Figure 1E). D-mannitol additionally regulates the osmolarity of the hydrogel to avoid osmolar stress on bioprinted cells, which require extracellular osmolarity to be as close as possible to the physiological one (~300 mOsm).

### 3.2. Characterization of the 3D Bioprinted Cervical Tumor Model

HeLa cells were 3D bioprinted in the alginate bioink supplemented with mannitol. It is known that HeLa cells embedded in an alginate matrix tend to form spheroids and could result in a good experimental model for drug testing [63]. However, an evaluation of parameters characterizing the spheroid population over time of culturing (such as cell survival rate, cell morphology, and spheroid dimensions) was lacking so far; this information is required when spheroids are used as cervical tumor models. Additionally, using immunofluorescence we evaluated the expression of cadherins, proteins involved in cell–cell adhesion. This information would be extremely valuable in view of SECM measurements of oxygen concentrations inside spheroids and drug absorption and diffusion in the three-dimensional matrix; in particular, the dimension of the spheroid is directly correlated with the extent of intra-spheroid oxygen gradients, with cell oxygen consumption, spheroid diameter and oxygen diffusion rate in the spheroid extracellular matrix all contributing to its establishment.

For the cervical tumor model establishment, a three-layer cube 1 cm × 1 cm of alginate bioink with 1.5 mm height was printed, and subsequently three lines of cell-embedded bioink were bioprinted within this prebuilt construct. Lines were 1 cm in length and they were 3 mm distant from each other, while holding a good filament uniformity (Figure 1F). 

Cell viability in the 3D samples was assessed by live/dead analysis at 3 h after biofabrication, to determine the influence of the printing process on cell viability, and at 24 h, 48 h, and 72 h after biofabrication, to determine the cell viability upon culturing into our 3D matrix. HeLa cells were affected by the printing procedure but not by the culturing in the bioprinted constructs, and the viability at 3h after printing was around 60%, while viability increased around 90% at 24 h, 48 h, and 72 h (Figure 2). After 72 h, as a consequence of the formation of spheroids and cell aggregates, it became more difficult to distinguish single cells with the live/dead standard procedure. 

**Figure 3:**
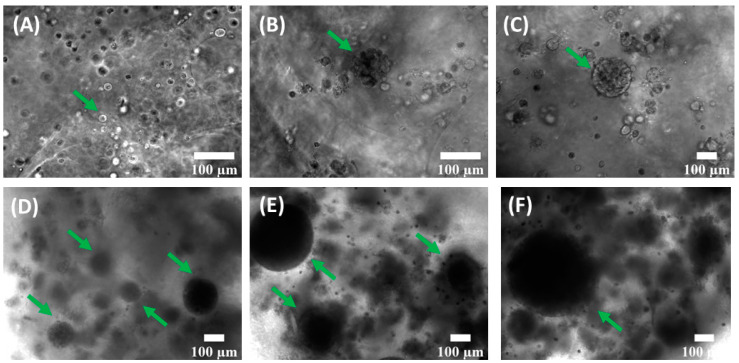
HeLa spheroid formation in a 3D alginate matrix, printed according to the optimized procedure, during four weeks of culture. (**A**) HeLa cell distribution and organization 1 day after printing; the green arrow indicates a single cell. (**B**) Representative example of cellular organization at 7 days after printing; the green arrow indicates an area where spheroid organization is starting. (**C**) 3D bioprinted matrix 10 days after printing: the green arrow indicates a HeLa spheroid. (**D**,**E**) 18 days (**D**) and 23 days (**E**) after printing; the green arrows indicate spheroids. HeLa spheroids are increasing, both in number and average dimensions. (**F**) HeLa spheroids and surrounding matrix 32 days after printing: a green arrow indicates a spheroid with a diameter of 470 μm; the degree of cellularization of the sample ulteriorly increases.

Due to the transparency of the bioink, these 3D models can be observed by optical microscopy to evaluate cell growth and the formation of spheroids. HeLa cells embedded in alginate bioink grew in a spheroid fashion, with the formation of spherical clusters of cells starting from duplication of a single cell. Spheroids appeared after a few days of culture and they grew over time, both in dimensions and number. We cultured the 3D sample for ~35 days, monitoring the dimensions and morphology of spheroids, as shown in Figure 3 (spheroids are indicated by the green arrows) and Figure S4. After 10 days, spheroids showed well-defined sphericity (as shown in Figure 3C), while immature spheroids, at ~7 days of culture, have mostly a grape-like shape, as depicted in Figure 3B. As can be seen from phase-contrast images, the degree of cellularization of the sample increased over the weeks, often preventing at later stages the observation of spheroid architecture by phase-contrast microscopy. Examples of spheroids and the surrounding matrix at 18, 23, and 32 days after printing are reported in Figure 3D–F. After 32 days of printing, several spheroids with a diameter of hundreds of micrometers could be observed (a spheroid with a diameter of 470 μm is shown in Figure 3F). 

**Figure 4:**
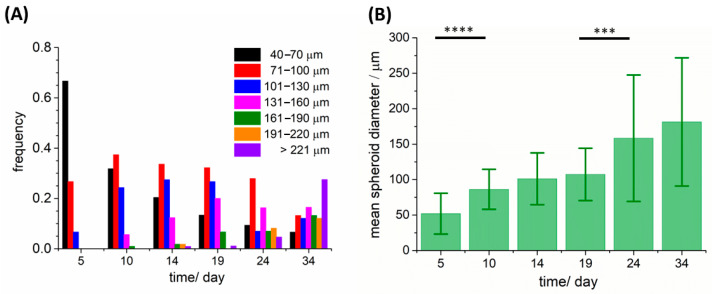
Analyses of HeLa spheroid diameter over several weeks (at day 5, 10, 14, 19, 24, 34). (**A**,**B**) Frequency distribution of spheroid diameter (**A**) and mean diameter in the spheroid population ((**B**), mean ± standard deviation) with increasing culturing time. The number of spheroids at each time point are reported as: day 5, 22 spheroids; day 10, 107 spheroids; day 14, 113 spheroids; day 19, 72 spheroids; day 24, 86 spheroids; day 34, 91 spheroids. Levels of statistical significance are calculated as detailed in Section 2.9 and are indicated as follow: ***, *p* < 0.001; ****, *p* < 0.0001.

The frequency distribution of spheroid diameters at different time points from the bioprinting is shown in Figure 4A; Figure 4B reports the mean spheroid diameters and the relative standard deviations as error bars at several timepoints, namely 5, 10, 14, 19, 24, and 34 days. The distribution widened over the weeks due to cell proliferation: during week 1 (day 5), spheroids mostly had a diameter ranging between 40 and 70 µm, while a few of them had a diameter ranging from 71 µm to 130 µm. From week 2 (day 10) to week 4 (day 24) there was a prevalence of spheroids with a diameter between 71 and 100 µm and the mean diameter value changed from 80 µm to 155 µm. At the end of week 2, the distribution was populated with some spheroids that had a diameter larger than 130 µm, and the frequency of spheroids with a lower diameter started to decline in favor of larger ones. At week 5, spheroids had diameter dimensions mostly in the range between 131 µm and 160 µm. The average diameter at week 5 was 175 µm, but one third of the total number of spheroids were bigger than 221 µm and the maximum value observed was around 500 µm, as shown in Figure 3F. 

**Figure 5:**
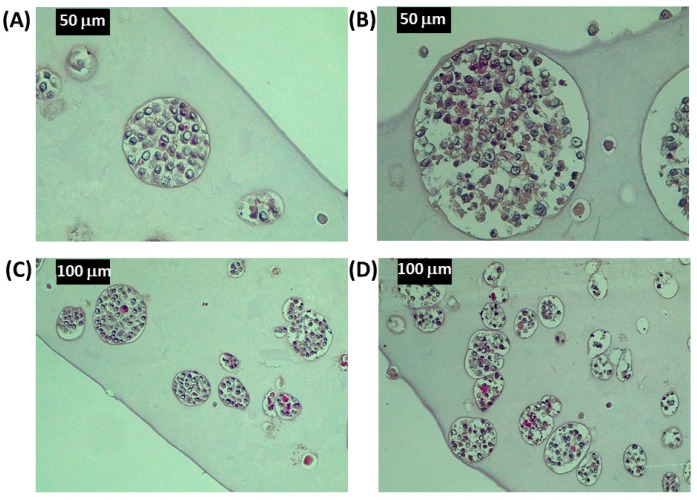
Histological images of HeLa spheroids in the 3D bioprinted alginate matrix. HE staining of HeLa spheroids in the alginate matrix after 11 (**A**,**C**) and 18 (**B**,**D**) days of culturing. Magnification factor: (**A**,**B**), 40×; (**C**,**D**), 20×.

The morphology of HeLa spheroids was further evaluated by hematoxylin–eosin (HE) staining, enabling the observation of cellular organization in the spheroid mass. Samples were observed after 11 and 18 days of culture (Figure 5): volumes of spheroids proved to be filled with cells and characterized by a spherical shape. After 18 days of culturing, the cells in the spheroids looked more shrunken and smaller than those at 11 days (Figure 5B). At longer culturing times, spheroids tended to merge, due to the increase in neighboring spheroids and the high cellularization of the matrix, as shown in Figure 5D. Empty spaces in the matrix are observed in the correspondence of the spheroids in fixed samples; this feature was due to histological procedures, which caused partial dehydration of samples.

**Figure 6:**
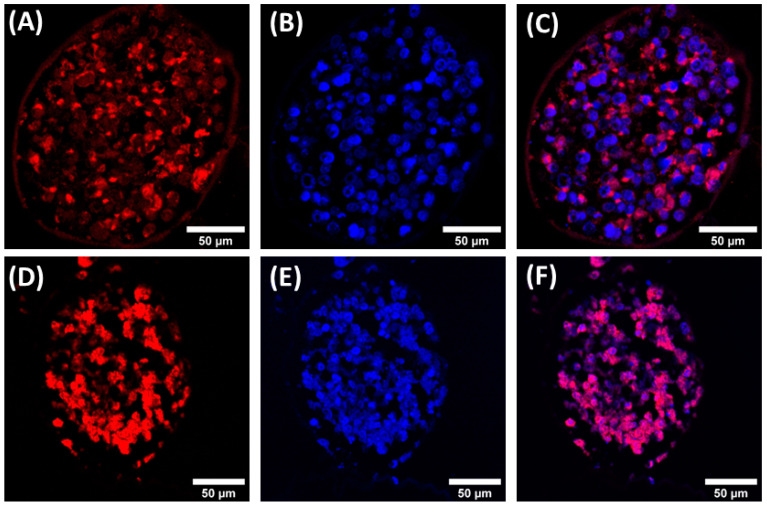
Confocal fluorescence images of Hela spheroids. E-cadherin immunolocalization (red, antibody conjugated with AlexaFluor 568) and nuclei staining (blue, DAPI) at 11 (**A**–**C**) and 18 (**D**–**F**) days of culturing. Colocalization by merging of the two channels at 11 (**C**) and 18 (**F**) days of culture.

The expression of E-cadherin, which cells typically employ to mediate cell–cell adhesion, was investigated using immunofluorescence. It would be of interest to report on the production of these molecules by the cells in our 3D bioprinted cellular microenvironment and to evaluate whether the production of these molecules is influenced by the presence of the tridimensional alginate structure, as the expression of this protein is involved in the formation of spheroids and tridimensional cellular aggregates [64,65]. If the 3D bioprinted microenvironment better represents the tumor microenvironment as compared with standard bidimensional cell cultures, then the expression of E-cadherin within the 3D HeLa spheroids should be higher as compared with 2D cultures; the results (Figure S3C,D) confirmed this expectation, as already documented in the literature for HeLa/HUVEC co-cultures [66]. HeLa spheroids at 11 days (Figure 6A–C) and 18 days (Figure 6D–F) after culturing are shown in Figure 6. Two spheroids of similar diameter (approximately 150 µm) were compared for E-cadherin production after 11 and 18 days of culturing. Our results confirm that E-cadherin is expressed during the formation of spheroids and its production qualitatively increases with longer culturing time (Figure 6C–F). 

### 3.3. Spatial Characterization by SECM of Oxygen Consumption in Cervical Tumor Models and Drug Diffusion in the 3D Matrix 

Phase-contrast microscopy and fluorescence confocal microscopy of 3D bioprinted HeLa cervical tumor models enabled the characterization of the dimensions of spheroids in the population, the organization of cells in the spheroid volume, and the expression of cellular membrane proteins which characterize cells cultured in three dimensions. In the developed alginate-based bioink, 3D bioprinted HeLa cells produced spheroids with a mean spheroid diameter increasing from approximately 50 µm after five days of culturing to 150 µm after three weeks. The average dimensions of the spheroid population influenced the spatial distribution of metabolites and nutrients in the 3D cellular object, shaping gradients of crucial metabolites such as, for example, oxygen. Scanning electrochemical microscopy was employed to measure oxygen concentration inside the spheroid volume and the diffusion kinetics of molecules in the three-dimensional matrix in which they were embedded. This biophysical characterization of the cervical tumor model could be extremely important for investigating the ability of the cervical model to reproduce key biophysical characteristics of tumor tissues, such as hypoxia and drug diffusion kinetics, in the tumor mass microenvironment.

Oxygen concentration was measured by employing disk-shaped platinum nanoelectrodes with active diameters of 200–500 nm. Originally, in these electrodes, the platinum wire is sealed and pulled into a quartz glass body with an outer glass diameter of 0.9 mm, resulting in a variable total diameter of the tip at the extremity that is around 15 µm. With a view to penetrating the spheroids without altering their spheroid structure, we set up a method to diminish the external tip diameter, by immersing about 1 cm of the probe in 48% hydrofluoric acid (see Materials and Methods section for details). The breakage of spheroids caused by tip penetration was thus prevented, and the delicate penetration of spheroids was achieved by employing sharp nanoelectrode tips. 

**Figure 7:**
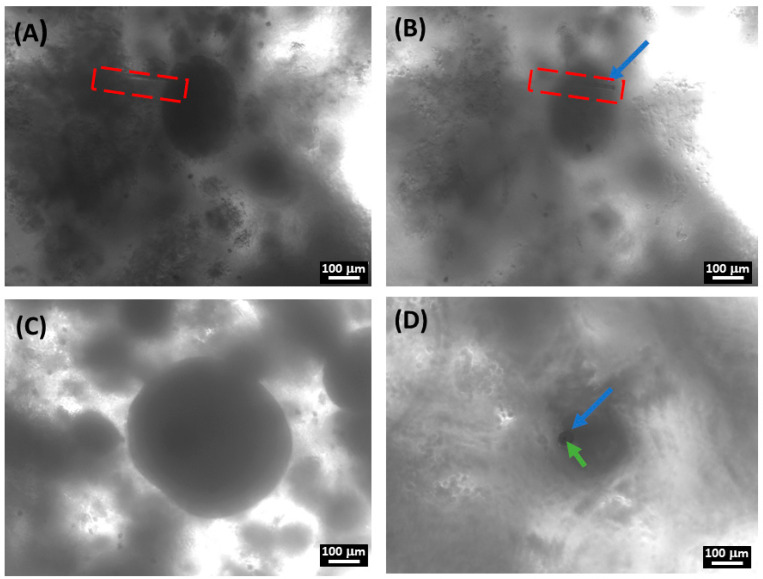
Phase-contrast images during oxygen content SECM measurements inside single spheroids. (**A**,**B**) Lateral penetration in the side of a spheroid with diameter of ~250 µm; before (**A**) and after (**B**) penetration. The nanoelectrode tip is indicated in (**B**) with a blue arrow; the optical focus is adjusted to resolve the nanoelectrode tip in the different locations. In (**A**,**B**) the nanoelectrode profile is contoured with a red dashed line. (**C**,**D**) Bright field images of vertical penetration inside spheroids with a diameter of ~450 µm; spheroid before (**C**) and after (**D**) penetration. The black dot indicated by the green arrow in (**D**) is the active part of the nanoelectrode; the grey hole indicated by the blue arrow is the upper part seen in perspective. The upper part of the nanoelectrode did not penetrate the spheroid; the optical focus was adjusted to make the nanoelectrode tip visible.

The coupling of SECM with a phase-contrast microscope enabled the monitoring and guiding of spheroid penetrations during the electrochemical measurements. We could measure oxygen concentration inside the spheroid volume in real time during penetration of the spheroids: Figure 7 shows representative images during the penetration steps. As already mentioned in the Introduction, spatially resolved measurements of oxygen content of spheroids was fundamental to characterize the presence and the extent of the hypoxic core, which is one of the main reasons for spheroids being considered good 3D models of real tumor masses. 

**Figure 8:**
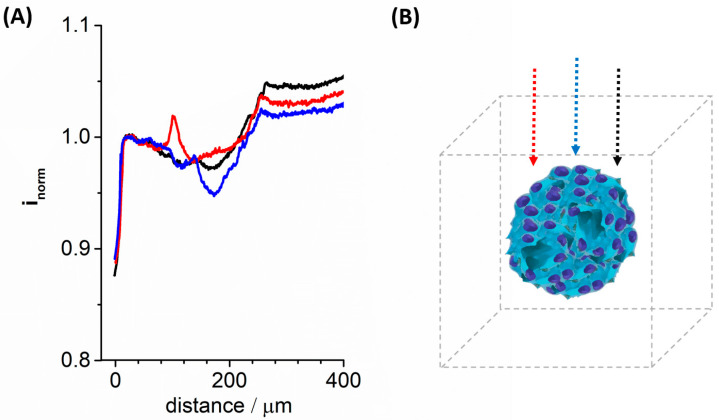
SECM measurements of oxygen concentration inside spheroids with a diameter of ~250 µm. (**A**) The three different measurements were made inside the same spheroids in adjacent sections. The blue curve is oxygen concentration measured in the longer spheroid diameter (blue arrow in panel (**B**)); black and red curves are measurements performed on more peripheral parts of the spheroid (black and red arrow, respectively, in the panel (**B**). Measurements were performed using a 200–500 nm Pt UME at −0.7 V vs. Ag/AgCl (KCl 3 M) with a scan rate of 10 μm/s in PBS solution. These measurements are representative of three measurements performed on three different spheroids. The current detected at the nanoelectrode has been normalized (i_norm_) with respect to that measured at a fixed distance from the bottom of the Petri dish and outside the spheroid, in this case this distance was 28 μm.

Figure 8 shows SECM vertical scans taken while penetrating spheroids and detecting oxygen at the nanoelectrode tips, by applying −0.6 V vs. the quasi-reference silver wire electrode. At this working potential, reduction of oxygen took place at the nanoelectrode tips and reduction currents were measured. As shown in Figure 8, oxygen reduction current decreased while the penetration occurred. The currents reached the minimum point in the center of the spheroid and then increased again while going further to the other side. We performed the scan at three different points within the same spheroid: one in the center of the spheroid (Figure 8, blue trace) and the other two at 20 μm on the right and 50 μm on the left side of the spheroid (Figure 8, red and black traces). Oxygen tension was expected to increase radially from the core to the outside of the spheroid [67]; the current measured in the center was consistently lower than the current measured in either of the two sides. As a consequence of the central chord being bigger, oxygen concentration decreased more steeply as a function of the overall distance from the spheroid surface when compared with the side chords. In the center of the spheroid, the current decreased for the most central chord about 57% more than the most external one (black curve).

These SECM measurements allowed for minimally invasive monitoring of cell metabolites in living spheroids with a sub-micrometric spatial resolution; thus, this unique and powerful technique permitted the characterization of the tridimensional microenvironment of 3D cell cultures. 

**Figure 9:**
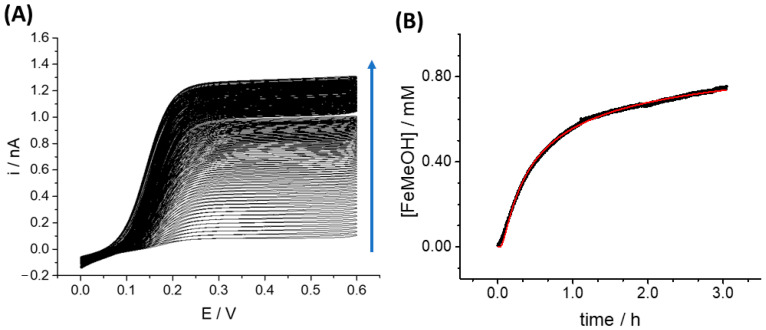
(**A**) Cyclic Voltammetries of FeMeOH diffusion in 3D bioprinted tissue. Measurement of FeMeOH diffusion in the 3D alginate sample followed by cyclic voltammetry over time. Oxidation current increased as FeMeOH concentration increased over time (the direction of the blue arrow indicates increasing time). (**B**) Diffusion coefficient curve of FeMeOH (black curve) in the alginate 3D sample obtained from repeated cyclic voltammetries; the best fitting (red curve) using the Equation (1) was calculated using OriginPro 9.1 software. Values of free parameters for the best fit are *C*_0_ = (1.705 ± 0.003) mM and *D* = (2.57 ± 0.02) cm^2^ s^−1^. The distance of the microelectrode from the nearest construct/solution boundary, *x*, was kept as x = 0.8 mm as a non-adjustable parameter during best fit. This value was measured from the SECM approach curve to the bottom of the Petri dish.

SECM was also employed to measure the diffusion of molecules in the printed alginate scaffold; these measurements allowed the characterization of diffusion phenomena in the 3D cell culture models that is crucial to understanding drug and metabolite activities in these models, which better mimic the tridimensional structure of real human tissues. We employed ferrocenemethanol (FeMeOH) as a model to demonstrate the potentiality of SECM to evaluate diffusion constants of a specific molecule of interest in the 3D matrix. FeMeOH is a redox compound, and its concentration can be easily detected by applying positive potentials to the Pt electrode; thus, when measuring FeMeOH oxidation occurring at the UME tip when positioned in the 3D bioprinted construct, these currents are directly proportional to the local concentrations of the molecule. Diffusion of the molecule in the alginate scaffold was measured using cyclic voltammetry (Figure 9A) performed continuously, starting at the moment of FeMeOH addition to the 3D construct growing medium (t = 0) up to reaching equilibrium of FeMeOH concentration in the matrix. The exact position of the electrode in the 3D construct could be controlled by the SECM probe approach curve. 

A current/time graph using the current values recorded in cyclic voltammetry in the anodic scan, at 0.4 V vs. quasi-reference Ag wire, was plotted to describe FeMeOH diffusion in the matrix. A zero concentration of FeMeOH was associated with the current value recorded just before the addition of FeMeOH to the growing medium, and the current value recorded at the equilibrium was relative to 1.5 mM FeMeOH. 

For the microelectrode, the stationary oxidation current is given by:

(1) $$i_{\left(t,\infty \right)}=4nFDCa$$

where *i_t,∞_* is the FeMeOH oxidation current recorded at the microelectrode; *n* is the number of electrons involved in the oxidation process (one for FeMeOH); *F* is the Faraday constant; *D* is the diffusion constant of FeMeOH in the medium; *C* is the concentration of FeMeOH; and *a* is the electrode tip radius. 

Given that the oxidation current is directly proportional to the concentration, a concentration/time graph could be obtained from the current/time graph, as shown in Figure 9B. This concentration/time graph was fitted using an equation derived from Fick’s law, already employed to fit diffusion of molecules in human tissues [68]:

(2) $$C_{\left(x,t\right)}=C_{0}\left(1-erf\left(\frac{x}{2\sqrt{Dt}}\right)\right)$$

where: *C*_(*x,t*)_ is the concentration of the molecules reduced or oxidized at the microelectrode tip at time *t* and position coordinate *x*; *x* is the distance of the electrode from the surface of the 3D sample (800 μm), estimated by carrying out an SECM Probe Approach Curve on the bottom of the dish after the measurement; *C*_0_ is the bulk concentration (called the boundary concentration) of the species detected at the microelectrode, i.e., 1.5 mM FeMeOH; *erf* is the error function; D is the diffusion constant of FeMeOH in the medium; and *t* is time.

Best fitting of FeMeOH oxidation currents extracted from cyclic voltammetries (see Materials and Methods section for a detailed description of the fitting procedure) returned a value of the diffusion coefficient of the molecule (*D*) equal to 2.6 × 10^−6^ cm^2^ s^−1^. This value is in the same order of magnitude of that reported in the literature for aqueous solutions, 7.8 × 10^−6^ cm^2^ s^−1^ [69], but is approximately three times smaller. This result is reasonable, as diffusion in a polymeric matrix as compared with pure water is expected to be slower; on this basis we concluded that the technique could be profitably used in three-dimensional cell cultures to study the diffusion of analytes and molecules in the 3D matrices and cellular drug absorption.

## 4. Conclusions and Future Perspectives

In this work, we present a 3D bioprinted cervical tumor model characterized by defined structural and dimensional characteristics of the 3D spheroids. Oxygen concentration gradients inside the spheroid masses was measured with high spatial resolution by SECM with non-destructive modalities by employing platinum nanoelectrodes; following two weeks of culturing we obtained a population of spheroids characterized by the hypoxic conditions that are typical of real tumor masses [70,71]. 

These results were pursued by first formulating an alginate bioink characterized by good extrudability and high filament uniformity that held high structural stability after printing. With this hydrogel formulation we developed a tridimensional cervical tumor model; HeLa cells in the matrix showed a high viability percentage (more than 90%). Moreover, these cells grew as spheroids and proliferated until 6 weeks, reaching diameter dimensions of several hundreds of microns. From confocal and microtome section data, we concluded that the spheroids were mainly round shaped and filled with living cells. We demonstrated that the obtained 3D cell culture model allowed the study of cells in a microenvironment similar to that in real, in vivo tissues. 

We employed scanning electrochemical microscopy to spatially resolve the oxygen concentration inside HeLa spheroids, by coupling the use of nanoelectrodes and real-time monitoring of spheroid penetration with phase-contrast microscopy. As well as for oxygen, it would be possible in the near future to investigate relevant cellular metabolites, to study the spatial distribution of these metabolites inside spheroid architecture. SECM was also employed to characterize how diffusional processes are modulated within these tridimensional cultures demonstrating that, in the near future, this approach could be used to evaluate drug penetration and distribution within the 3D bioprinted model, mimicking the diffusion of anticancer compounds in tumor masses. 

Among other scanning probe techniques, atomic force microscopy (AFM) is capable of accurately determining the morphological maps of a scanned sample [61]; the combination of AFM with SECM could be a powerful tool, with the aim of subtracting background signals due to morphological characteristics of the sample [62]. 

We finally conclude that there is an urgent need for characterization technologies for tridimensional models, and we think this work relevantly contributes in this direction.

## Data Availability

All data in this study can be requested from the corresponding author S.R., stefania.rapino3@unibo.it.

## Supplementary Materials

The following supporting information can be downloaded at: https://www.mdpi.com/article/10.3390/cancers15041327/s1, Figure S1: 3D bioprinted grid with the optimized sterile bioink; Figure S2: schematic representation of measurements of FeMeOH diffusion into 3D bioprinted constructs and of oxygen concentration in HeLa spheroids by SECM; Figure S3: confocal fluorescence images of bidimensional and tridimensional HeLa cultures; Figure S4: nanoelectrode tip dimension compared with single HeLa cells in the 3D construct, imaged by phase-contrast microscopy.

## References

[1] Intlekofer A.M., Finley L.W.S. (2019). Metabolic Signatures of Cancer Cells and Stem Cells. Nat. Metab..

[2] Faubert B., Solmonson A., DeBerardinis R.J. (2020). Metabolic reprogramming and cancer progression. Science.

[3] Bergers G., Fendt S.M. (2021). The Metabolism of Cancer Cells during Metastasis. Nat. Rev. Cancer.

[4] Hanahan D., Weinberg R.A. (2011). Hallmarks of Cancer: The next Generation. Cell.

[5] Junttila M.R., De Sauvage F.J. (2013). Influence of Tumour Micro-Environment Heterogeneity on Therapeutic Response. Nature.

[6] Quail D.F., Joyce J.A. (2013). Microenvironmental Regulation of Tumor Progression and Metastasis. Nat. Med..

[7] Rodrigues J., Heinrich M.A., Teixeira L.M., Prakash J. (2021). 3D In Vitro Model (R) Evolution: Unveiling Tumor–Stroma Interactions. Trends Cancer.

[8] Langer E.M., Allen-petersen B.L., King S.M., Kendsersky N.D., Turnidge M.A., Kuziel G.M., Riggers R., Samatham R., Taylor S., Jacques S.L. (2019). Modeling Tumor Phenotypes In Vitro with Three-Dimensional Bioprinting. Cell Rep..

[9] Jensen C., Teng Y. (2020). Is It Time to Start Transitioning From 2D to 3D Cell Culture?. Front. Mol. Biosci..

[10] Duval K., Grover H., Han L.-H., Mou Y., Pegoraro A.F., Fredberg J., Chen Z. (2017). Modeling Physiological Events in 2D vs. 3D Cell Culture. Physiology.

[11] Ravi M., Paramesh V., Kaviya S.R., Anuradha E., Paul Solomon F.D. (2015). 3D Cell Culture Systems: Advantages and Applications. J. Cell Physiol..

[12] Myungjin Lee J., Mhawech-Fauceglia P., Lee N., Cristina Parsanian L., Gail Lin Y., Andrew Gayther S., Lawrenson K. (2013). A Three-Dimensional Microenvironment Alters Protein Expression and Chemosensitivity of Epithelial Ovarian Cancer Cells in Vitro. Lab. Investig..

[13] Åkerlund H.E., Broglie J.J., Adcock A.F., Yang L. (2014). Three-Dimensional Cell Culture Systems and Their Applications in Drug Discovery and Cell-Based Biosensors. Assay Drug Dev. Technol..

[14] Hirschhaeuser F., Menne H., Dittfeld C., West J., Mueller-Klieser W., Kunz-Schughart L.A. (2010). Multicellular Tumor Spheroids: An Underestimated Tool Is Catching up Again. J. Biotechnol..

[15] Rankin E.B., Giaccia A.J. (2016). Hypoxic Control of Metastasis. Science.

[16] Lee P., Chandel N.S., Simon M.C. (2020). Cellular Adaptation to Hypoxia through Hypoxia Inducible Factors and Beyond. Nat. Rev. Mol. Cell Biol..

[17] Hammarlund E.U., Flashman E., Mohlin S., Licausi F. (2020). Oxygen-Sensing Mechanisms across Eukaryotic Kingdoms and Their Roles in Complex Multicellularity. Science.

[18] Griffith L.G., Swartz M.A. (2006). Capturing Complex 3D Tissue Physiology in Vitro. Nat. Rev. Mol. Cell Biol..

[19] Garde A., Sherwood D.R. (2021). Fueling Cell Invasion through Extracellular Matrix. Trends Cell Biol..

[20] Chaudhuri O., Cooper-white J., Janmey P.A., Mooney D.J., Shenoy V.B. (2020). Effects of Extracellular Matrix Viscoelasticity on Cellular Behaviour. Nature.

[21] Jiang Y., Zhang H., Wang J., Liu Y., Luo T., Hua H. (2022). Targeting Extracellular Matrix Stiffness and Mechanotransducers to Improve Cancer Therapy. J. Hematol. Oncol..

[22] Eble J.A., Niland S. (2019). The Extracellular Matrix in Tumor Progression and Metastasis. Clin. Exp. Metastasis.

[23] Ng W.L., Chua C.K., Shen Y.F. (2019). Print Me An Organ! Why We Are Not There Yet. Prog. Polym. Sci..

[24] Ozbolat I.T., Hospodiuk M. (2016). Current Advances and Future Perspectives in Extrusion-Based Bioprinting. Biomaterials.

[25] Li X., Liu B., Pei B., Chen J., Zhou D., Peng J., Zhang X., Jia W., Xu T. (2020). Inkjet Bioprinting of Biomaterials. Chem. Rev..

[26] Ng W.L., Lee J.M., Zhou M., Chen Y.W., Lee K.X.A., Yeong W.Y., Shen Y.F. (2020). Vat Polymerization-Based Bioprinting-Process, Materials, Applications and Regulatory Challenges. Biofabrication.

[27] Moroni L., Boland T., Burdick J.A., De Maria C., Derby B., Forgacs G., Groll J., Li Q., Malda J., Mironov V.A. (2018). Biofabrication: A Guide to Technology and Terminology. Trends Biotechnol..

[28] Hospodiuk M., Dey M., Sosnoski D., Ozbolat I.T. (2017). The Bioink: A Comprehensive Review on Bioprintable Materials. Biotechnol. Adv..

[29] Lin T.E., Rapino S., Girault H.H., Lesch A. (2018). Electrochemical Imaging of Cells and Tissues. Chem. Sci..

[30] Bartolini L., Malferrari M., Lugli F., Zerbetto F., Paolucci F., Pelicci P.G., Albonetti C., Rapino S. (2018). Interaction of Single Cells with 2D Organic Monolayers: A Scanning Electrochemical Microscopy Study. ChemElectroChem.

[31] Malferrari M., Ghelli A., Roggiani F., Valenti G., Paolucci F., Rugolo M., Rapino S. (2019). Reactive Oxygen Species Produced by Mutated Mitochondrial Respiratory Chains of Entire Cells Monitored Using Modified Microelectrodes. ChemElectroChem.

[32] Soldà A., Valenti G., Marcaccio M., Giorgio M., Pelicci P.G., Paolucci F., Rapino S. (2017). Glucose and Lactate Miniaturized Biosensors for SECM-Based High-Spatial Resolution Analysis: A Comparative Study. ACS Sens..

[33] Rapino S., Marcu R., Bigi A., Soldà A., Marcaccio M., Paolucci F., Pelicci P.G., Giorgio M. (2015). Scanning Electro-Chemical Microscopy Reveals Cancer Cell Redox State. Electrochim. Acta.

[34] Polcari D., Dauphin-Ducharme P., Mauzeroll J. (2016). Scanning Electrochemical Microscopy: A Comprehensive Review of Experimental Parameters from 1989 to 2015. Chem. Rev..

[35] Zhang J., Zhu T., Lang J., Fu W., Li F. (2020). ScienceDirect Electrochemistry Recent Advances of Scanning Electrochemical Microscopy and Scanning Ion Conductance Microscopy for Single-Cell Analysis. Curr. Opin. Electrochem..

[36] Bergner S., Vatsyayan P., Matysik F.M. (2013). Recent Advances in High Resolution Scanning Electrochemical Microscopy of Living Cells—A Review. Anal. Chim. Acta.

[37] Borghese R., Malferrari M., Brucale M., Ortolani L., Franchini M., Rapino S., Borsetti F., Zannoni D. (2020). Structural and Electrochemical Characterization of Lawsone-Dependent Production of Tellurium-Metal Nanoprecipitates by Photosynthetic Cells of *Rhodobacter Capsulatus*. Bioelectrochemistry.

[38] Malferrari M., Becconi M., Rapino S. (2019). Electrochemical Monitoring of Reactive Oxygen/Nitrogen Species and Redox Balance in Living Cells. Anal. Bioanal. Chem..

[39] Nebel M., Grützke S., Diab N., Schulte A., Schuhmann W. (2013). Visualization of Oxygen Consumption of Single Living Cells by Scanning Electrochemical Microscopy: The Influence of the Faradaic Tip Reaction. Angew. Chem. Int. Ed..

[40] De Zio S., Becconi M., Soldà A., Malferrari M., Lesch A., Rapino S. (2023). Glucose Micro-Biosensor For Scanning Electrochemical Microscopy Characterization of Cellular Metabolism in Hypoxic Microenvironments. Bioelectrochemistry.

[41] Sun P., Laforge F.O., Abeyweera T.P., Rotenberg S.A., Carpino J., Mirkin M.V. (2008). Nanoelectrochemistry of Mammalian Cells. Proc. Natl. Acad. Sci. USA..

[42] Clausmeyer J., Schuhmann W. (2016). Nanoelectrodes: Applications in Electrocatalysis, Single-Cell Analysis and High-Resolution Electrochemical Imaging. TrAC-Trends Anal. Chem..

[43] Zhang X.W., Qiu Q.F., Jiang H., Zhang F.L., Liu Y.L., Amatore C., Huang W.H. (2017). Real-Time Intracellular Measurements of ROS and RNS in Living Cells with Single Core–Shell Nanowire Electrodes. Angew. Chem. Int. Ed..

[44] Clausmeyer J., Actis P., López Córdoba A., Korchev Y., Schuhmann W. (2014). Nanosensors for the Detection of Hydrogen Peroxide. Electrochem. Commun..

[45] Mukomoto R., Nashimoto Y., Terai T., Imaizumi T., Hiramoto K., Ino K., Yokokawa R., Miura T., Shiku H. (2020). Oxygen Consumption Rate of Tumour Spheroids during Necrotic-like Core Formation. Analyst.

[46] Zhao L., Shi M., Liu Y., Zheng X., Xiu J., Liu Y., Tian L., Wang H., Zhang M., Zhang X. (2019). Systematic Analysis of Different Cell Spheroids with a Microfluidic Device Using Scanning Electrochemical Microscopy and Gene Expression Profiling. Anal. Chem..

[47] Zheng X.T., Li C.M. (2012). Single Cell Analysis at the Nanoscale. Chem. Soc. Rev..

[48] Plodinec M., Loparic M., Monnier C.A., Obermann E.C., Zanetti-Dallenbach R., Oertle P., Hyotyla J.T., Aebi U., Bentires-Alj M., Lim R.Y.H. (2012). The Nanomechanical Signature of Breast Cancer. Nat. Nanotechnol..

[49] Malferrari M., Turina P., Francia F., Mezzetti A., Leibl W., Venturoli G. (2015). Dehydration Affects the Electronic Structure of the Primary Electron Donor in Bacterial Photosynthetic Reaction Centers: Evidence from Visible-NIR and Light-Induced Difference FTIR Spectroscopy. Photochem. Photobiol. Sci..

[50] Northcutt L.A., Suarez-Arnedo A., Rafat M. (2020). Emerging Biomimetic Materials for Studying Tumor and Immune Cell Behavior. Ann. Biomed. Eng..

[51] Freeman F.E., Kelly D.J. (2017). Tuning Alginate Bioink Stiffness and Composition for Controlled Growth Factor Delivery and to Spatially Direct MSC Fate within Bioprinted Tissues. Sci. Rep..

[52] Schwab A., Levato R., D’Este M., Piluso S., Eglin D., Malda J. (2020). Printability and Shape Fidelity of Bioinks in 3D Bioprinting. Chem. Rev..

[53] Schindelin J., Arganda-Carreras I., Frise E., Kaynig V., Longair M., Pietzsch T., Preibisch S., Rueden C., Saalfeld S., Schmid B. (2012). Fiji: An Open-Source Platform for Biological-Image Analysis. Nat. Methods.

[54] Abdel Aziz I., Malferrari M., Roggiani F., Tullii G., Rapino S., Antognazza M.R. (2020). Light-Triggered Electron Transfer between a Conjugated Polymer and Cytochrome C for Optical Modulation of Redox Signaling. iScience.

[55] Munaz A., Vadivelu R.K., St. John J., Barton M., Kamble H., Nguyen N.T. (2016). Three-Dimensional Printing of Biological Matters. J. Sci. Adv. Mater. Devices.

[56] Zhang Y., Hu G., Liu Y., Wang J., Yang G., Li D. (2022). Suppression and Utilization of Satellite Droplets for Inkjet Printing: A Review. Processes.

[57] Bucatariu S.M., Constantin M., Varganici C.D., Rusu D., Nicolescu A., Prisacaru I., Carnuta M., Anghelache M., Calin M., Ascenzi P. (2020). A New Sponge-Type Hydrogel Based on Hyaluronic Acid and Poly(Methylvinylether-Alt-Maleic Acid) as a 3D Platform for Tumor Cell Growth. Int. J. Biol. Macromol..

[58] Nii T., Makino K., Tabata Y. (2019). A Cancer Invasion Model Combined with Cancer-Associated Fibroblasts Aggregates Incorporating Gelatin Hydrogel Microspheres Containing a P53 Inhibitor. Tissue Eng. Part C Methods.

[59] Nii T., Makino K., Tabata Y. (2020). Three-Dimensional Culture System of Cancer Cells Combined with Biomaterials for Drug Screening. Cancers.

[60] Singh S., Ray L.A., Shahi Thakuri P., Tran S., Konopka M.C., Luker G.D., Tavana H. (2020). Organotypic Breast Tumor Model Elucidates Dynamic Remodeling of Tumor Microenvironment. Biomaterials.

[61] Marcuello C., Frempong G.A., Balsera M., Medina M., Lostao A. (2021). Atomic Force Microscopy to Elicit Conformational Transitions of Ferredoxin-Dependent Flavin Thioredoxin Reductases. Antioxidants.

[62] Paiva T.O., Schneider A., Bataille L., Chovin A., Anne A., Michon T., Wege C., Demaille D. (2022). Enzymatic activity of individual bioelectrocatalytic viral nanoparticles: dependence of catalysis on the viral scaffold and its length. Nanoscale.

[63] Zhao Y., Yao R., Ouyang L., Ding H., Zhang T., Zhang K., Cheng S., Sun W. (2014). Three-dimensional printing of Hela cells for cervical tumor model in vitro. Biofabrication.

[64] Smyrek I., Mathew B., Fischer S.C., Lissek S.M., Becker S., Stelzer E.H.K. (2019). E-Cadherin, Actin, Microtubules and FAK Dominate Different Spheroid Formation Phases and Important Elements of Tissue Integrity. Biol. Open.

[65] (1991). Masatoshi Takeichi Cadherin Cell Adhesion Receptors as a Morphogenetic Regulator. Science.

[66] Lee J.M., Park D.Y., Yang L., Kim E.J., Ahrberg C.D., Lee K.B., Chung B.G. (2018). Generation of Uniform-Sized Multicellular Tumor Spheroids Using Hydrogel Microwells for Advanced Drug Screening. Sci. Rep..

[67] Langan L.M., Dodd N.J.F., Owen S.F., Purcell W.M., Jackson S.K., Jha A.N. (2016). Direct Measurements of Oxygen Gradients in Spheroid Culture System Using Electron Parametric Resonance Oximetry. PLoS ONE.

[68] Tuchin V., Genina E., Larin K. (2008). Measurement of Glucose Diffusion Coefficients in Human Tissues. Handbook of Optical Sensing of Glucose.

[69] Park J.H., Thorgaard S.N., Zhang B., Bard A.J. (2013). Single Particle Detection by Area Amplification: Single Wall Carbon Nanotube Attachment to a Nanoelectrode. J. Am. Chem. Soc..

[70] Ast T., Mootha V.K. (2019). Oxygen and Mammalian Cell Culture: Are We Repeating the Experiment of Dr. Ox?. Nat. Metab..

[71] Keeley T.P., Mann G.E. (2019). Defining Physiological Normoxia for Improved Translation of Cell Physiology to Animal Models and Humans. Physiol. Rev..

