# Supplementary material for: Nano-Electrochemical Characterization of a 3D Bioprinted Cervical Tumor Model
Source: Cancers (Basel). 2023 Feb 19;15(4):1327. doi: 10.3390/cancers15041327 (PMC9954750; doi:10.3390/cancers15041327)
Supplement: Supplementary file 1 [file cancers-15-01327-s001.zip › cancers-2143268-supplementary.pdf]

cancers

MDPI

Supplementary Materials: Nano-Electrochemical Characterization of a 3D Bioprinted Cervical Tumor Model

Maila Becconi, Simona De Zio, Francesco Falciani, Marzia Santamaria, Marco Malferrari and Stefania Rapino

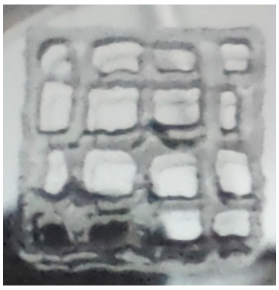

Figure S1. 3D bio-printed grid with the final sterile bioink, formulated with 2.5% w/v alginate, 3.3% w/v D-Mannitol, 0.19% w/v $CaCl_2$ in bi-distilled water.

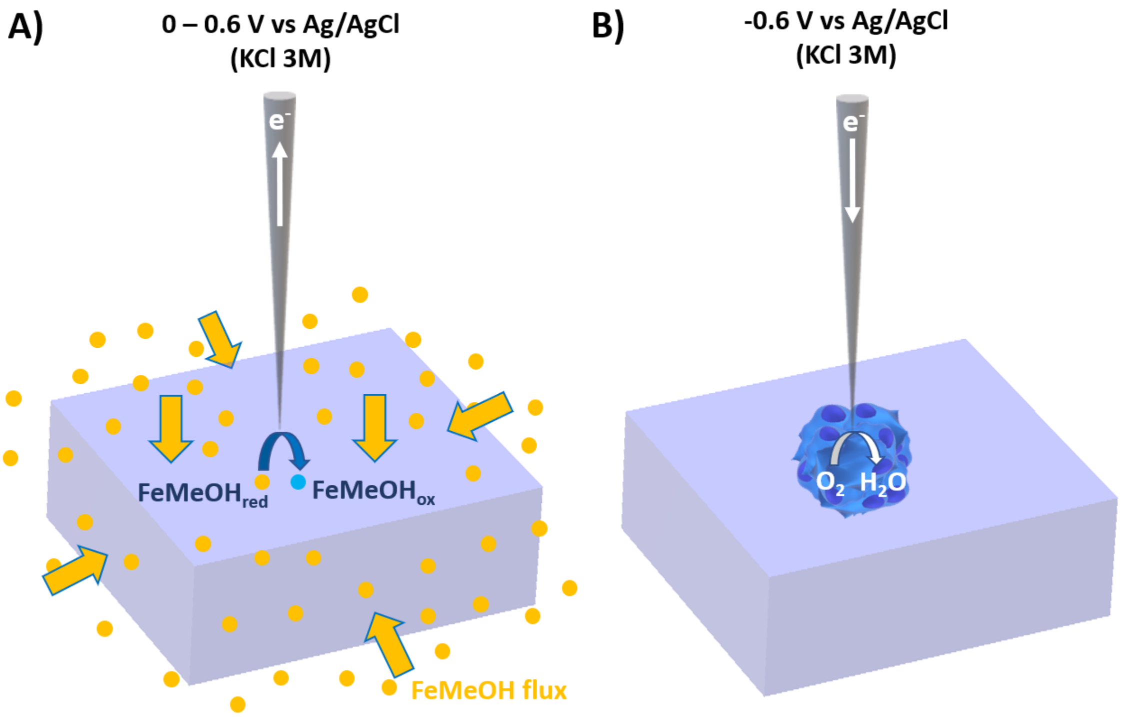

Figure S2. Schematic representation of SECM detection of (**A**) FeMeOH diffusion into 3D bio-printed constructs (**B**) and measurements of oxygen concentrations in HeLa spheroids.

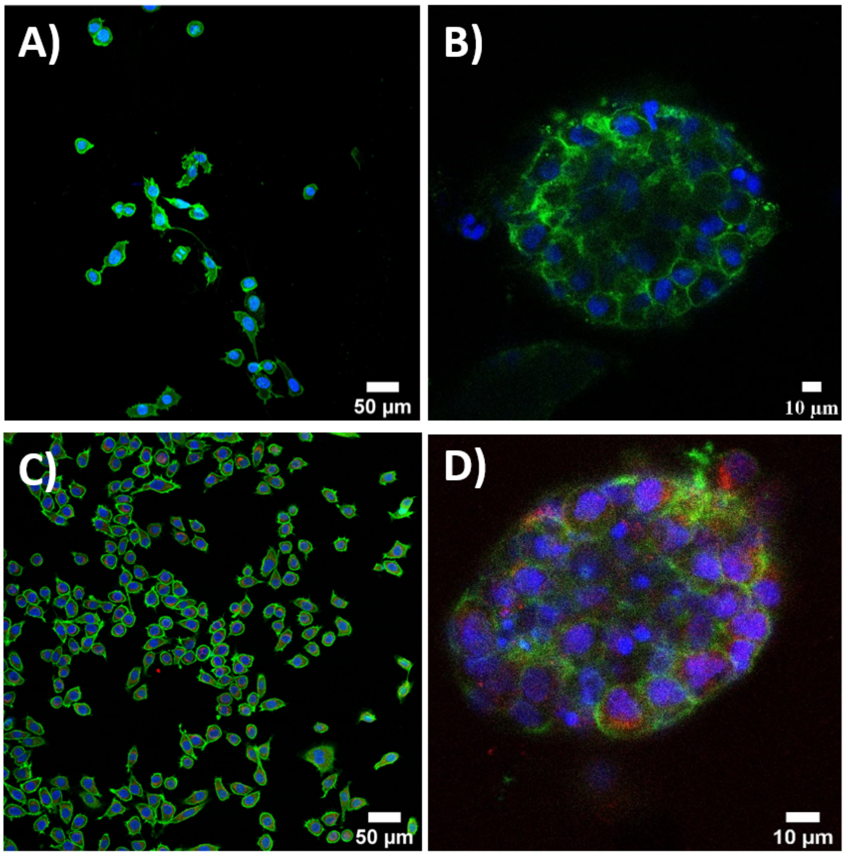

Figure S3. Confocal images of HeLa morphological structure in 2D and 3D. **(A,C)** Bidimensional and **(B,D)** tridimensional cultures of HeLa cells. Nuclei staining with DAPI and cytoskeleton staining with Phalloidin-FITC are shown in blue and green, respectively. E-cadherin expression is showed in (**C)** and (**B)** with red staining.

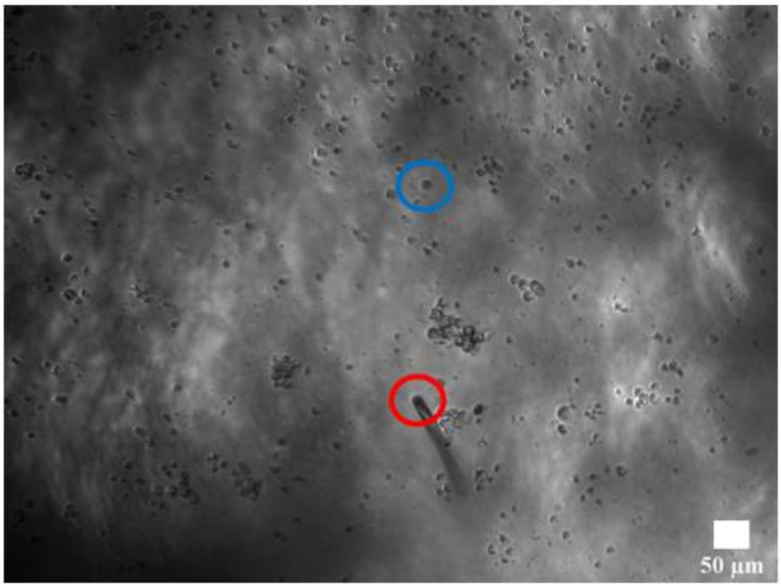

Figure S4. Comparison between the dimension of the employed nanoelectrode and a single HeLa **cell**: the red circle indicates the tip and the blue circle indicates a single HeLa cell growing in 3D alginate matrix.
